# Are prescribing doctors sensitive to the price that their patients have to pay in the Spanish National Health System?

**DOI:** 10.1186/1472-6963-11-333

**Published:** 2011-12-08

**Authors:** Beatriz González López-Valcárcel, Julián Librero, Gabriel Sanfélix-Gimeno, Salvador Peiró

**Affiliations:** 1 Departamento de Métodos Cuantitativos. Universidad de Las Palmas de Gran Canaria. Las Palmas de Gran Canaria, Spain; 2 Centro Superior de Investigación en Salud Pública (CSISP). Valencia, Spain; 3 Instituto Aragonés de Ciencias de la Salud. Zaragoza, Spain

## Abstract

**Background:**

This study aims to design an empirical test on the sensitivity of the prescribing doctors to the price afforded for the patient, and to apply it to the population data of primary care dispensations for cardiovascular disease and mental illness in the Spanish National Health System (NHS). Implications for drug policies are discussed.

**Methods:**

We used population data of 17 therapeutic groups of cardiovascular and mental illness drugs aggregated by health areas to obtain 1424 observations ((8 cardiovascular groups * 70 areas) + (9 psychotropics groups * 96 areas)). All drugs are free for pensioners. For non-pensioner patients 10 of the 17 therapeutic groups have a reduced copayment (RC) status of only 10% of the price with a ceiling of €2.64 per pack, while the remaining 7 groups have a full copayment (FC) rate of 40%. Differences in the average price among dispensations for pensioners and non-pensioners were modelled with multilevel regression models to test the following hypothesis: 1) in FC drugs there is a significant positive difference between the average prices of drugs prescribed to pensioners and non-pensioners; 2) in RC drugs there is no significant price differential between pensioner and non-pensioner patients; 3) the price differential of FC drugs prescribed to pensioners and non-pensioners is greater the higher the price of the drugs.

**Results:**

The average monthly price of dispensations to pensioners and non-pensioners does not differ for RC drugs, but for FC drugs pensioners get more expensive dispensations than non-pensioners (estimated difference of €9.74 by DDD and month). There is a positive and significant effect of the drug price on the differential price between pensioners and non-pensioners. For FC drugs, each additional euro of the drug price increases the differential by nearly half a euro (0.492). We did not find any significant differences in the intensity of the price effect among FC therapeutic groups.

**Conclusions:**

Doctors working in the Spanish NHS seem to be sensitive to the price that can be afforded by patients when they fill in prescriptions, although alternative hypothesis could also explain the results found.

## Background

In National Health Systems (NHS) with public funding prescribing physicians can be considered as double agents acting as patients' advocates but also as society's gatekeepers of resource use. Public healthcare organizations -health authorities and managers- put pressure on physicians to control pharmaceutical spending, and more so the lower the patient co-payment. According to economic theory [1], doctors are patients' agents and as such prescribe treatments that maximize the utility, effectiveness and quality of care in the face of the patients' preferences and economic and other restrictions. The completeness of this agency relationship is the subject of theoretical (multiple models of physicians' behaviour, and on the doctor-patient relationship) and empirical debate. From a policy perspective, to investigate whether prescribing doctors are sensitive to the price paid by their patients (related or not with the agency relationship) is an interesting topic because it enables foreseeing of the impact and effectiveness of alternative pharmaceutical cost-containment measures. If NHS doctors prescribe cheaper, clinically equivalent medicines to patients that have a co-payment, this may be suggestive of the proper functioning of the doctor-patient agency relationship, but it could also be a symptom of an ex-post moral hazard in the sense that when patients do not bear the cost of treatment they receive the most expensive one, not necessarily the most cost-effective [2].

The Spanish NHS is particularly suited to the empirical study of this issue. One relevant characteristic of the Spanish NHS is that Spain is divided into 17 autonomous regions, known as "Autonomous Communities", with a high degree of self-government, including the responsibility for health care. Each Spanish regional government manages a network of hospital and primary healthcare centres which provide free inpatient care and consultations to about 97% of the population. These regional networks are organized into healthcare areas of variable size (usually between 150,000 and 250,000 inhabitants) with one acute public hospital and several primary healthcare centres serving the population resident in a delimited geographical territory [3]. Care in these services is free of charge, with coverage extending to substantial pharmaceutical benefits: all medicines prescribed to pensioners (eligible because of age, retirement from work or disability) and underprivileged collectives are free of charge. Relatives under the care of pensioners are also included in the exemptions from payment status. The remaining population, referred to in this study as "non-pensioners" (in Spain, known as "active") pay for only part of the costs of medicines through a co-payment system with the following characteristics: the general co-payment rate is 40% of the cost of the medication but in order to avoid charging patients with unaffordable payments over long time periods, long-term treatments for chronic conditions are usually charged at only 10%, with a ceiling of €2.64 (in 2010) per package (in Spain drugs are dispensed in commercial packages, not in unitary doses customized for each patient, and a separate prescription form must be filled out for each package). The copayment status of each drug is regulated by the Spanish Ministry of Health and is mandatory for all Autonomous Regions. From now on, we will refer to the drugs charged at the 40% general rate as regular prescriptions or full-copayment (FC) drugs, and we will call those drugs with 10% co-payment (up to €2.64) reduced co-payment (RC) drugs.

Family physicians in the Spanish NHS are paid by salary with small variable incentives -different between regions- depending on achievement of goals (including prescription of generic or equivalent but cheaper drugs) and workload. The drugs prescribed by NHS doctors are dispensed in private pharmacies at fixed prices regulated by the Spanish government. The list of drugs (brand names and presentations) that may be covered by NHS has very few exclusions. The drugs financed, prices and copayments are identical in all Autonomous Communities, and discounts are not allowed. There is a reference pricing system for pharmaceuticals when the patent has expired, but while in other countries the patient is allowed to pay the difference between the reference price and the price of the prescribed drug if the latter is higher, in Spain the reference price system excludes drugs outside the reference threshold from public coverage, and has resulted in uniformity of prices among brand name drugs (with patent expired) and generics [4].

All Spanish regions have the same regulation on prices and copayments. The price of a particular drug presentation is the same throughout the country. As prices of brand drugs (and generics) within a given therapeutic class of drugs differ, geographical differences in the observed average prices of prescribed drugs for a particular therapeutic class come from compositional differences: a region where many doctors prescribe generics or cheaper drugs will show lower average prices. As a result, in the Spanish NHS the average price discrepancy for a therapeutic group between two health areas is due to price differentials in the shopping cart of dispensed drugs, which depends on the choice between more expensive (i.e. brand name drugs with patents in force) or cheaper drugs (i.e. generics) in the corresponding therapeutic group.

The double differential copayment between groups of patients (non-pensioners vs. pensioners) and between drugs (40% copayment vs. 10% up to €2.64) allows the designing of a test on the intensity of doctors' sensitivity to the price paid by their patients. If doctors do not take into account the copayment that patients have to face, they would be expected to prescribe at the same average price for non-pensioners and pensioners. If the physician were an agent acting on behalf of the Administration, he would be mindful of public expenditure. Apart from that, if the physician had incentives to limit the total cost of prescribed medications, such as by being a factor in the calculation of the physicians' variable pay, then the average price of drugs could even be higher for non-pensioners than for pensioners in that doctors would be more concerned to prescribe low cost drugs to pensioners because they are charged to the NHS; and if doctors were concerned about expenses to the patient's pocket, then the average price of regular prescriptions for non-pensioners would be less than the average price for pensioners within the same drug group, but no difference, or a small difference, would be expected for the average price of RC drugs.

This study aims to design an empirical test on the presence and intensity of doctors' sensitivity to the price paid by their patients, and to implement it with testable hypotheses applied to the data of primary care dispensations for cardiovascular disease and mental illness in the NHS in Spain. We will also discuss the implications for drug policies.

## Methods

### Population

The units of analysis were the 1424 observations derived from multiplying the 17 therapeutic groups by the healthcare areas [(8 cardiovascular groups * 70 areas) + (9 psychotropic groups * 96 areas) = 1424] participating in two previous research projects designed to describe small area variations in drug utilization. From the first project, on cardiovascular drugs, we use data on dispensation and expenditure for 8 therapeutic groups in 70 healthcare areas from 5 autonomous communities for 2005 [5]. From the second project, on psychotropic drugs, we obtained data on 9 therapeutic groups in 96 healthcare areas from 8 Autonomous Communities for 2006 [6]. In 2006, these areas had between 8,528 and 846,253 inhabitants, and globally the study population of the second project was 24.9 million inhabitants (18,96 million non-pensioners and 5,94 million pensioners), representing 56.4% of the Spanish population (47.7% in the first project).

### Sources of data

The data used in this study came from the claims that the regions' pharmacies submit to the respective Autonomous Community's Health Departments on a monthly basis. The data used in our study are propierty of the respective Regional Health Departments and are not publicly avalaible. Data were requested for the research group and the Regional Health Departments transferred them with the corresponding permission to use in the *Grupo de Investigación en Utilización de Medicamentos en el Sistema Nacional de Salud *(Drug Utilization in the Spanish National Health System Research Group) projects. Among other data, these claims include information about the drug dispensed (brand name, formulation, dose, number of units per package, price) and the coverage characteristics of the patient: pensioner (free of charge) or non-pensioner (under the co-payment scheme). The claims do not include any patient information about age, sex, diagnostic features or reason for prescription. Since drug packages do not necessarily contain the same quantities of drugs, prescribed quantities were transformed into defined daily doses (DDDs), the amount that corresponds to the average maintenance dose per day for a medication used in its principal indication in adults, as established by the World Health Organization's Collaborating Centre for Drug Statistics Methodology [7,8].

### Pharmacological groups

The selection of pharmacological groups was opportunistic, depending on the previous projects [5,6]. We excluded three groups comprising virtually only one medicine or marketed at an almost identical price (doxazosin, digitalis, spironolactone) because in those cases average prices could not be different. Table 1 describes the 17 pharmacological subgroups included according to the Anatomical Therapeutic Chemical (ATC) classification system. Ten of them are RC drugs and the remaining 7 are regular FC drugs. In both conditions, cardiovascular and mental illness, medicines of both co-payment groups coexist, allowing us to contrast our hypothesis. Fixed-dose combinations were assigned following the ATC criteria. Some of the drugs included (the anti-platelets agents different to aspirin, such as clopidogrel) are subject to prior authorization requirements that include, among other, age-related criteria.

**Table 1:** Description of pharmacological groups.

Pharmacological group	ATC codes	Description	Copayment
**Cardiovascular groups**			

Beta-blockers	C07A, C07B, C07C, C07D, C07F	Beta blocking agents, plain and combined with thiazides, other diuretics, and other antihypertensives.	RC

ACEIs & ARBs	C09A, C09B, C09C, C09D	ACE inhibitors, plain and combinations, Angiotensin II antagonists plain and combinations	RC

Diuretics	C03A, C03B, C03C, C03EA, C03EB	Low-ceiling diuretics, thiazides or other, high-ceiling diuretics, and diuretics and potassium-sparing agents in combination.	RC

Nitrates	C01DA	Organic nitrates.	RC

Ca^++ ^channel blockers	C08C, C08D	Selective calcium channel blockers with mainly vascular effects and selective calcium channel blockers with direct cardiac effects.	RC

Antiplatelet drugs	B01AC04, B01AC06	Clopidogrel and acetylsalicylic acid (in dose of 100 mg)	FC

Flavonoids	C05CA03, C05CA04, C05CA05	Diosmin, troxerutin, hidrosmin.	FC

Statines	C10AA	HMG-CoA reductase inhibitors (statines)	FC

**Psychotropic drugs**			

SSRIs	N06AB, N06AX	Selective serotonin reuptake inhibitors and other antidepressants	RC

Typical antipsychotics (1st generation)	N05AA, N05AB, N05AC, N05AD, N05AF, N05AG, N05AK	Phenothiazines with aliphatic side-chain, piperazine structure or piperidine structure, butyrophenone derivatives, thioxanthene derivatives and diphenylbutylpiperidine derivatives	RC

Atypical Antipsychotics (2nd generation)	N05AE, N05AH, N05AL, N05AX	Indole derivatives, diazepines, oxazepines, thiazepines, oxepines, benzamides and other antipsychotics	RC

MAOIs	N06AF, N06AG	Non-selective monoamine oxidase inhibitors, monoamine oxidase A inhibitors	RC

Lithium	N05AN	Lithium	RC

Anti-dementia drugs	N06DA, N06DX	Anticholinesterases, and other anti-dementia drugs	FC

Hypnotics	N05CA, N05CB, N05CC, N05CE, N05CD, N05CF, N05CM, N05CX	Barbiturates, plain and combinations, aldehydes and derivatives, penzodiazepine derivatives, piperidinedione derivatives, benzodiazepine related drugs, other hypnotics and sedatives, hypnotics and sedatives in combination, excl. barbiturates	FC

Anxiolytics	N05BA, N05BB, N05BC, N05BD, N05BE, N05BX	Benzodiazepine derivatives, diphenylmethane derivatives, carbamates, dibenzo-bicyclo-octadiene derivatives, azaspirodecanedione derivatives, and other anxiolytics	FC

Psychostimulants	N06BA, N06BX	Centrally acting sympathomimetics and other psychostimulants and nootropics	FC

ATC: Anatomical Therapeutic Chemical classification system; ACEI: angiotensin-converting enzyme inhibitors; ARB: angiotensin receptor blockers; SSRIs: Selective Serotonin Reuptake Inhibitors; MAOIs: Monoamine oxidase inhibitors. Copayment: FC if non-pensioner patients pay a copayment of 40%, RC if non-pensioner patients pay a reduced rate of 10% with a ceiling of €2.64 (all treatments are free for pensioners)

### Testable hypotheses

NHS doctors, as agents of their patients, look at the out-of-pocket cost of treatment for patients. Therefore, we expect no differences (or lower differences) in average prices of RC drugs prescribed to pensioner patients and to non-pensioner patients, while we expect a positive average price differential for FC drugs, becoming greater the higher the price is. The specific testable hypotheses include: H1A) In FC drugs (40% co-payment for non-pensioners) there is a significant difference between the average prices of drugs prescribed to pensioners and non-pensioners; H1B) There is no significant price differential between pensioners and non-pensioners in RC drugs, since both groups of patients are virtually exempt from payment; H2) The price differential for FC drugs is greater the higher the price of the drug is, after accounting for the clinical possibilities of substitution among drugs within a therapeutic subgroup.

### Analysis

From the DDD dispensed and the corresponding expenditure, we estimated the monthly average price of the DDD dispensed to pensioners and to non-pensioners, and the differential price between pensioner and non-pensioner patients for each geographical area and pharmacological subgroup (defined in Table 1). After a descriptive analysis and bivariate tests of equality of means, we carried out multivariate multilevel regressions for the price differences between pensioners and non-pensioners for therapeutic group j in area i, controlling for the type of co-payment (FC vs. RC), with random effects for the therapeutic group. Statistical comparisons among models were based on the deviance [9]. We estimated three models: Model 1, with random effects of the therapeutic group in the intercept, tests hypothesis H1A y H1B, and Models 2 and 3 test H2. Model 3 generalizes Model 2, by including random effects of the therapeutic group not only in the intercept but also in the slope of the price. In Model 2, FC drugs have a price differential between pensioner and non-pensioner patients that increases linearly with price, while RC drugs do not have such a differential between either groups of patients because of the ceiling for RC medicines. Models were specified as:

$$\begin{array}{c} M1:DP_{ij}=\beta_{1}FC_{j}+\beta_{2}RC_{j}+u_{j0}+e_{ij}\\ M2:DP_{ij}=\beta_{0}+\beta_{3}price_{ij}*FC_{j}+u_{j0}+e_{ij}\\ M3:DP_{ij}=\beta_{0}+\beta_{3}price_{ij}*FC_{j}+u_{j0}+u_{j3}price_{ij}*FC_{j}+e_{ij} \end{array}$$

where DP is the monthly price differential between pensioner and non-pensioner patients, FC is a dummy variable = 1 for regular drugs with co-payment of 40%, RC is a dummy = 1 for the reduced co-payment drugs, and price is the monthly price of a standard treatment (DDD) in euros. We used Maximum Likelihood for estimating the models, with Stata v11.0 (College Station, TX, USA).

## Results

Table 2 shows, for each pharmacological group, the quantities dispensed, the monthly average price for pensioner and non-pensioner patients, the differential price between them and the percentage of areas in which the average price for pensioners is higher than for non-pensioners. Only in 5 of the 17 groups was the average monthly price of prescriptions higher for non-pensioners, and only one of them (anxiolytics) is FC. Four of the 7 FC groups show differentials higher than €1 between pensioner and non-pensioner patients. Only 1 in 10 RC groups shows a differential higher than €1 between pensioner and non-pensioner patients.

**Table 2:** Price and price differential between prescriptions for pensioners and non-pensioners by pharmacological group.

	Copayment	DDD/1000/year		Price month		Price	%
				
		Pensioners	Non-pensioners	Pensioners	Non-pensioners	**Diff**.	Areas
**Cardiovascular groups**							

Beta-blockers	RC	46.08	6.12	9.72	8.78	0.90	80.00

ACEIs & ARBs	RC	326.47	29.99	14.34	13.81	0.54	68.57

Diuretics	RC	113.47	6.74	5.66	5.40	0.26	41.43

Nitrates	RC	53.21	0.82	11.90	12.02	-0.12	40.00

Ca^++^channel blockers	RC	109.35	6.73	17.85	15.89	1.96	98.57

Antiplatelet drugs	FC	98.74	4.96	14.34	11.45	2.89	94.29

Flavonoids	FC	44.06	3.45	7.95	8.46	0.50	50.00

Statines	FC	187.18	17.70	19.84	18.29	1.54	98.57

**Psychotropic drugs**							

SSRIs	RC	98.72	21.99	26.32	26.11	0.21	34.38

Typical antipsych.	RC	5.35	0.25	8.61	8.82	-0.21	39.58

Atypical antipsych.	RC	18.71	2.23	125.24	130.57	-5.33	18.75

MAOIs	RC	0.10	0.02	15.79	14.43	1.35	42.71

Lithium	RC	1.02	0.28	5.62	5.80	-0.18	52.08

Anti-dementia	FC	18.40	0.19	82.18	27.99	54.18	100.00

Hypnotics	FC	69.04	6.04	3.20	3.05	0.14	84.38

Anxiolytics	FC	124.34	19.66	4.46	4.74	-0.28	6.24

Stimulants	FC	0.53	0.61	57.78	52.86	4.92	76.04

ACEI: angiotensin-converting enzyme inhibitors; ARB: angiotensin receptor blockers; SSRIs: Selective Serotonin Reuptake Inhibitors; MAOIs: Monoamine oxidase inhibitors. Copayment: FC if non-pensioners pay a copayment of 40%, RC if non-pensioners pay a reduced co-payment of 10% with a ceiling of €2.64; DDD/1000/year: Daily defined doses dispensed per 1000 people and year; Price: cost of one month of treatment; Price differences: between the monthly cost of treatment between pensioners and non-pensioners; % areas: % of areas with monthly costs of treatment higher in pensioners than in non-pensioners.

**Figure 1:**
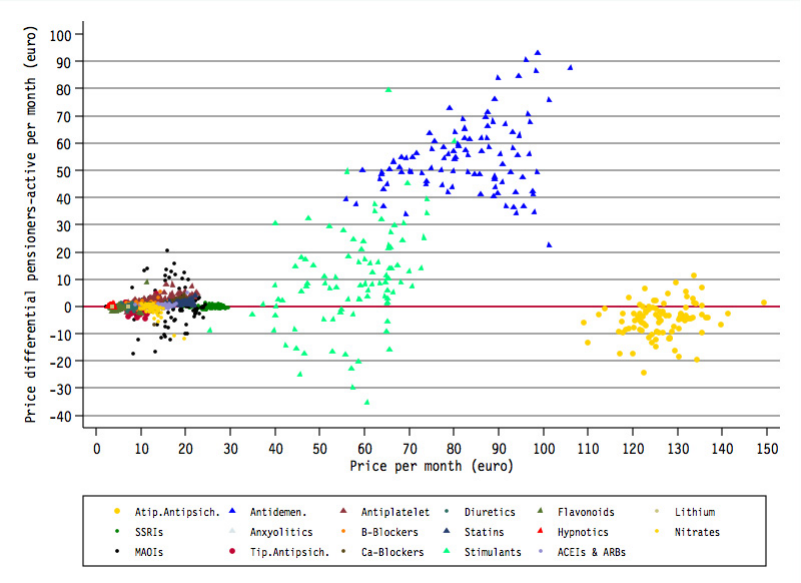
**Differential of prices between pensioner and non-pensioner patients by copayment status and therapeutic group**. ACEI: angiotensin-converting enzyme inhibitors; ARB: angiotensin receptor blockers; SSRIs: Selective Serotonin Reuptake Inhibitors; MAOIs: Monoamine oxidase inhibitors. Full co-payment drugs are represented as triangles and reduced copayment drugs are represented as circles.

Figure 1 shows the relationship between the monthly price differential between pensioners and non-pensioners, and the monthly price by copayment status. Two clusters of RC drugs can be appreciated, one of a lower price (less than €1 per day) and another of a higher price (stimulants, anti-dementia drugs and atypical antipsychotics). According to our hypothesis, FC drugs (triangles in Figure 1) show a positive price differential that increases linearly with price, particularly in the case of stimulants and anti-dementia drugs. On the contrary, and also as expected according to our hypothesis, RC drugs (circles in Figure 1) show cost differentials distributed around the zero value, indicating that pensioners get more expensive drugs than non-pensioners in some areas, while the opposite happens in other areas.

The average monthly price of RC cardiovascular drugs is higher than that of FC drugs (€14.4 vs. €12.4, p < 0.005), but we did not find significant differences among the prices of RC and FC psychotropic drugs. The average price differential among prescriptions to pensioners and non-pensioners for RC drugs was €-0.43 per month, while this differential for FC drugs was €10.8. The standard deviation of the latter is high (€21.6). Only in 16 observations out of the 1424 was a negative price differential higher than €5 observed. All the 16 are psycho-stimulants. We found variability among areas in drugs' average monthly price, particularly for flavonoids (coefficient of variation: 0.42). On the other hand, anxiolytics (low price) and atypical antipsychotics (high price) show homogeneous prices among areas with a coefficient of variation around 5%.

Table 3 shows the results of multilevel regression models M1 to M3. According to the M1 model, the monthly average price of dispensations to pensioners and non-pensioners is the same for RC drugs (the RC coefficient is non significant). On the other hand, the FC dummy is significant, indicating that pensioners get more expensive dispensations than non-pensioners (a difference of €9.74 per month). The M2 model estimated a positive and significant effect of the drug price on the differential price between pensioner and non-pensioner patients. For FC drugs, each additional euro of the drug price increases the differential by nearly half a euro (0.492). The random effect of the pharmacological groups is less intense than in the M1 model, but clearly significant with a withinclass correlation of 48.9%. According to the deviance test, the M2 model was superior to the M1 model. The M3 model, a generalization of M2 with random effects on the price coefficient, does not improve the adjustment over M2, and the random effect on the slope was non significant, indicating the absence of differences among the FC pharmacological groups in the intensity of the price effect.

**Table 3:** Factors explaining the monthly price differential between pensioners and non-pensioners dispensations.

Models		M1	M2	M3
Variables fixed part: estimated coefficients and significance	FC	9.74*	--	--
	
	RC	-0.32	--	--
	
	Price*FC	--	0.4920**	0.4273**
	
	Intercept	--	-1.71	-1.44

Random effects	Withinclass correlation	77.2%	48.9%	61.6%^#^
	
	Var(U_0_)	143.7376	36.5447	58.3749
	
	Var(U_3_)	--	---	0.0147
	
	Var(e)	42.3655	38.1651	37.7826

Deviance (-2 Log L)		9399.3	9230.4	9228.8

Multilevel models.

n = 1413. *p < 0.05; **p < 0.01; ^# ^Estimated for the monthly average (€12.0). FC if non-pensioners pay a copayment of 40%, RC if non-pensioners pay a reduced co-payment of 10% with a ceiling of €2.64.

## Discussion

The results of this study show that dispensation data (a feasible proxy of doctor prescriptions) are compatible with our hypothesis on physicians' behaviour. The more expensive a drug is, the higher the price differential is between dispensations to pensioner and non-pensioner patients if non pensioners pay a 40% copayment, but this differential is inexistent in drugs with a reduced copayment, suggesting that Spanish NHS physicians are sensitive to the price that their patients have to pay.

There are three pharmacological FC groups for which the effect is clearer, because they include drugs of high price and drugs of low price: antiplatelet agents (clopidogrel vs. acetylsalicylic acid), statins (atorvastatin vs. simvastatin and other statins) and anti-dementia drugs (anticholinesterases and memantine vs. ginkgo biloba). In these groups, doctors seem to differentiate prices and predominantly prescribe low price drugs to non-pensioners and high price drugs to pensioners. Therefore, these groups have a positive price differential for pensioners in almost all healthcare areas. However, doctors do not seem to distinguish prices for non-expensive or low-price drugs. For them, the differential effect is negligible. One possible explanation for this behaviour is that physicians only have an approximate knowledge of drug prices. This explanation is consistent with a review of physicians' awareness of drug prices, showing a low cost accuracy (31% of estimates were within 20% or 25% of the true cost, and fewer than 50% were accurate by any definition of cost accuracy) [10]. This result is also consistent with a previous study for Spain. Spanish family physicians were asked about the price of well-known drugs. They estimated correctly (with an interval of 25% around the real price) in 41% of cases. This study also suggests that physicians tend to neglect price differences between products of identical composition [11].

In contrast with the growth in the literature about (co)payment effects on healthcare service utilization (quantities), empirical studies about the effects on prices are scarce. Our study is consistent with those scarce antecedents, confirming that the selection of the prescribed drug is influenced by the price that the patient pays (or co-pays). Even in Japan, where physicians sell medicines to the patients and have incentives to obtain higher margins with more expensive drugs, one study in antihypertensive drugs found that "physicians are willing to give up one dollar of their profit in order to reduce the co-payment of non-elderly patients by 28 cents" [12]. In Sweden, another study concluded that physicians prescribed less expensive drugs (generics) to patients that had to pay for them [2]. On the other hand, several studies using qualitative methods [13] or surveys [14-18] have reported that physicians claim to consider out-of-pocket costs a more important issue when prescribing than the cost for the organization or for society (although physicians -occasionally in the same studies- declare their awareness of drug costs and that discussing the cost of treatment with patients is very uncommon) [15,17-19]. Notably, the most likely strategy used to assist patients burdened by their out-of-pocket costs is to switch the patient from a brand name to a generic drug [20,21].

Going beyond the agency relationship, doctors' price sensitivity to their patients' copayment scheme may be the result of different causal mechanisms. First, pensioners are older and probably sicker than non-pensioners, and occasionally some expensive drugs could be more appropriate for patients at higher risk (i.e., clopidogrel has a lower risk of gastrointestinal bleeding than aspirin and could be a better alternative for elderly people). Second, the pharmaceutical industry exerts strong promotional pressures on doctors to prescribe new, more expensive drugs with a patent in force. It is possible that marketing strategies focus -at least in some cases- on older patients, contributing to higher prices (although these hypotheses cannot explain the absence of differences in drugs with reduced copayment). Third, in Spain general practitioners maintain the prescription of medicines that have been indicated by specialists (so-called "induced prescription"). Specialists have different prescription patterns (with more innovative and expensive drugs) and, also, treat more complex -and probably, older- patients. Pensioners could be more exposed to the "induced prescription" phenomenon than non-pensioners and therefore receive more expensive prescriptions. Finally, if patients with co-payment do not pick up (selectively) the most expensive prescriptions from pharmacies, we would be facing a problem of patients' price sensitivity instead of physicians' sensitivity to the price that can be afforded by patients.

### Limitations

Apart from contributing to the scarce literature on this issue, our study has certain other strengths. We work with population data and we include all the dispensations for selected therapeutic groups for two common conditions. The study also has several limitations. First, pensioners are very different from non-pensioners in terms of age, disease patterns and their severity, and these differences could justify differences in the choice of drugs and, therefore, in the average price for pensioners and non-pensioners. The ecological nature of the data does not allow consideration of all the factors that influence medical prescriptions (disease and its severity, other accompanying health conditions, possible contraindications or interactions with other drugs that the person is taking alongside, and so on). But in our study, average price discrepancy is measured within specific and relatively homogeneous therapeutic groups. In most of these therapeutic groups, evidence of the superiority of one drug over others in terms of higher price rarely exists (i.e. atorvastatin vs. simvastatin, ARBs vs. ACEIs, brand name vs. generic drugs, one atypical antipsychotic vs. another atypical antipsychotics, and so on) and, with some exceptions, there are no clinical reasons for the systematic use of high price drugs in pensioners and low price drugs or generics in non-pensioners. Nevertheless, in some cases the therapeutic groups are more heterogeneous, including medicines with different indication profiles (i.e. antiplatelet drugs or atypical antipsychotics). For those groups including some medicines aimed at young people and others aimed at older patients, the price differential could be a compositional effect not related with doctors' sensitivity to patient costs (i.e. requirements for the prior authorization of clopidogrel consider age over 65 as a criterion; because people over 65 are mainly pensioners with no copayment, we could find a compositional effect in this therapeutic class).

Second, more severe patients may use stronger doses of the same drug. Although strong-dose packages have a higher price, the DDD metric oscillates between flat pricing (equal for all presentations without considering the number of units or their strength) and monotonic pricing (the price of the DDD decreases with increasing units or doses of the presentation). Because pensioners usually consume presentations with higher doses and more units per presentation, the DDD price is artificially lower in this group, underestimating the copayment effect and undervaluing the intensity of doctors' sensitivity to patient costs.

Third, the Spanish regulation of prices and copayments does not consider a reduced contribution for fixed-dose combinations, even if both (or more) drugs of the combination separately have this consideration. As disaggregate data of prescriptions within each group were not available for this study, groups with fixed-dose combinations suffer a miss-classification bias (i.e. a third of the dispensations of inhibitors of the renin-angiotensin system group were fixed-dose combinations subject to a general copayment of 40%, but they were classified as RC drugs). This bias also underestimates the copayment effect and doctors' sensitivity to patient costs. Nonetheless, Model 3 does not show differences among pharmacological groups, suggesting that doctors' sensitivity to patient costs is independent of the medicines used.

Finally, and probably the most important limitation in our study, our conceptual framework attributes decisions on prescriptions to physicians but uses dispensations (prescriptions filled out) as a proxy of prescriptions issued (which also includes unfilled prescriptions). The patients' ability to influence prescription decisions to cheaper drugs is irrelevant to the agency theory (in fact, if the agency relationship was complete, the decision would always reflect the patients' preferences), but unfilled prescriptions overestimate doctors' sensitivity to patient cost effects, especially if patients do not pick up the most costly medicines from the pharmacy. Some studies in the United States have shown that the drug abandonment rate increases as the out-of-pocket expenses increase [22]. Although the generalization of these studies to the Spanish setting is uncertain, probably both behaviours (patients' price sensitivity and physicians' sensitivity to the price that can be afforded by their patients) occur at the same time and both contribute to the price differences between co-payment schemes detected in our study. The nature of our data (dispensation, not prescription) does not permit the estimation of the contribution of each factor to the price differences found.

### Implications

The policy implications of our findings for cost-containment are diverse. First of all, specific measures addressed to patients (i.e. the use of reference prices as avoidable copayments for pensioner and non-pensioner patients) could be effective measures for increasing doctors' prescription of cheaper and generic drugs. Second, cost-containment policies could benefit from a better knowledge of drug prices among physicians. Also, if our results are related with the agency relationship, physicians' incentives to switch expensive medicines to cheaper equivalents or generics should considerer physicians' beliefs on the clinical value of the cheaper ones relative to more expensive drugs. Nevertheless, we need broader and deeper studies on cost-containment pharmaceutical policies, and specifically on the agency relationship between physicians and their patients [23]. And, we evidently need better data about costs and reasons for prescription [23,24].

## Conclusions

The main finding of our study is that patients receive cheaper medicines when they have to pay the 40% copayment. Although part of this effect can be due to unfilled prescriptions (patients' price sensitivity) and other confounding factors, these results suggest that doctors are sensitive to the out-pocket costs that their patients have to bear, and that copayments, apart from the well-known effects on quantities dispensed, also influence the unitary costs of dispensed drugs.

## Competing interests

Most researchers of the IUM-SNS Group work in institutions under the Spanish Autonomous Communities' Health Departments. These Departments, the participating institutions and the organizations funding the research project do not necessarily share the contents of this manuscript.

## Authors' contributions

BGLV conceived the idea, did the data analyses and wrote the first draft of the manuscript. JL, GSG and SP prepared the databases, participated in conceiving the study design, data analyses and interpretation of the data. GSG coordinated the data collection. All members of the IUM-SNS Group were involved in data collection, review and data depuration, and discussing relevant issues. All authors have been involved in the discussions and writing of the paper and approved the final submitted version.

## Pre-publication history

The pre-publication history for this paper can be accessed here:

http://www.biomedcentral.com/1472-6963/11/333/prepub
